# DSP1 and DSP4 Act Synergistically in Small Nuclear RNA 3′ End Maturation and Pollen Growth^1^

**DOI:** 10.1104/pp.19.00231

**Published:** 2019-06-21

**Authors:** Xuepiao Pu, Chunmei Meng, Weili Wang, Siyu Yang, Yuan Chen, Qingjun Xie, Bin Yu, Yunfeng Liu

**Affiliations:** a State Key Laboratory for Conservation and Utilization of Subtropical Agro-Bioresources, College of Life Science and Technology, Guangxi University, Nanning, Guangxi 530004, China; b Life Sciences Institute, Guangxi Medical University, Nanning, Guangxi 530021, China; c Plant Gene Expression Center, U.S. Department of Agriculture–Agricultural Research Service and Department of Plant and Microbial Biology, University of California, Berkeley, California 94710; d State Key Laboratory for Conservation and Utilization of Subtropical Agro-Bioresources, Guangdong Provincial Key Laboratory of Plant Molecular Breeding, South China Agricultural University, Guangzhou, Guangdong 510642, China; e Center for Plant Science Innovation and School of Biological Sciences, University of Nebraska, Lincoln, Nebraska 68588-0660

## Abstract

Small nuclear RNAs (snRNAs) play essential roles in spliceosome assembly and splicing. Most snRNAs are transcribed by the DNA-dependent RNA polymerase II (Pol II) and require 3′-end endonucleolytic cleavage. We have previously shown that the Arabidopsis (*Arabidopsis thaliana*) Defective in snRNA Processing 1 (DSP1) complex, composed of at least five subunits, is responsible for snRNA 3′ maturation and is essential for plant development. Yet it remains unclear how DSP1 complex subunits act together to process snRNAs. Here, we show that DSP4, a member of the metallo-β-lactamase family, physically interacts with DSP1 through its β-Casp domain. Null *dsp4-1* mutants have pleiotropic developmental defects, including impaired pollen development and reduced pre-snRNA transcription and 3′ maturation, resembling the phenotype of the *dsp1-1* mutant. Interestingly, *dsp1-1 dsp4-1* double mutants exhibit complete male sterility and reduced pre-snRNA transcription and 3′-end maturation, unlike *dsp1-1* or *dsp4-1*. In addition, Pol II occupancy at snRNA loci is lower in *dsp1-1 dsp4-1* than in either single mutant. We also detected miscleaved pre-snRNAs in *dsp1-1 dsp4-1,* but not in *dsp1-1* or *dsp4-1*. Taken together, these data reveal that DSP1 and DSP4 function is essential for pollen development, and that the two cooperatively promote pre-snRNA transcription and 3′-end processing efficiency and accuracy.

## Abstract (precis)

DSP1 and DSP4 function synergistically in pollen development and promote pre-snRNA transcription and 3′-end processing efficiency and accuracy.

Small nuclear RNAs (snRNAs; Hadjiolov et al., 1966), a class of noncoding RNAs, are the basal components of the spliceosome and play essential roles in pre-mRNA splicing (Black et al., 1985; Ray et al., 1997; Guo et al., 2009). Their biogenesis involves transcription and subsequent processing steps. In Arabidopsis (*Arabidopsis thaliana*), the DNA-dependent RNA polymerase II (Pol II) synthesizes the primary snRNA transcripts (pre-snRNAs) U1, U2, U4, U5, and U12, but not U6, which is transcribed by Pol III (Carbon et al., 1987; Vankan and Filipowicz, 1988). Following transcription, pre-snRNAs are subjected to endonucleolytic cleavage at specific sites to remove the RNA fragment transcribed beyond the 3′-end of mature snRNAs.

In metazoans, snRNA 3′-end maturation requires the Integrator Complex (INT; Baillat et al., 2005). INT contains at least 14 subunits (Baillat et al., 2005; Chen and Wagner, 2010), associates with the C-terminal domain of the largest subunit of Pol II, and depends on transcription for complex formation. It cotranscriptionally cleaves pre-snRNAs upstream of the required 3′-box RNA motif (Uguen and Murphy, 2003, 2004; Baillat et al., 2005;Chen and Wagner, 2010). Integrator Subunit 11 (INT11) is a putative member of the metallo-β-lactamase (MBL)/β-CASP family of RNA endonucleases, is homologous to the cleavage and polyadenylation specificity factor 73 kD (CPSF73) that catalyzes pre-mRNA 3′-end cleavage, and is considered to be the enzyme that cleaves pre-snRNAs at the 3′ end. INT9 is also a CPSF73 homolog, but lacks key amino residues critical for endonuclease activity (Baillat et al., 2005; Li et al., 2016). INT9 and INT11 form a heterodimer through their C-terminal domains and this interaction is critical for the 3′-end processing efficiency of pre-snRNAs (Albrecht and Wagner, 2012; Wu et al., 2017). Besides snRNA 3′ maturation, INT also functions in other biological processes, including transcription termination of mRNAs, maturation of some viral-derived microRNAs, prevention of viral infection, biogenesis of enhancer RNAs, and dynein localization at the nuclear envelope (Jodoin et al., 2013; Gardini et al., 2014; Stadelmayer et al., 2014; Lai et al., 2015; Skaar et al., 2015; Xie et al., 2015; Li et al., 2016). Consistent with the importance of these functions, mutations in INT subunits often result in embryo lethality (Hata and Nakayama, 2007; Rutkowski and Warren, 2009; Ezzeddine et al., 2011; Kapp et al., 2013).

Plant snRNA 3′ maturation does not depend on transcription, although it requires a 3′ box motif whose sequence differs from its metazoan counterpart (Connelly and Filipowicz, 1993). In plants, the Defective in snRNA Processing 1 (DSP1) complex and the CPSF complex, both of which contain CPSF73-I, are responsible for the 3′-end cleavage of pre-snRNAs and pre-mRNAs, respectively. The DSP1 complex is composed of at least four additional subunits, DSP1 to DSP4 (Liu et al., 2016). Disruption of DSP1, DSP3, DSP4, or CPSF73-I, but not DSP2, impairs pre-snRNA processing, resulting in increased accumulation of pre-snRNAs. However, as in *int* mutants, the accumulation of mature snRNAs is not altered in the *dsp* mutants. Interestingly, Pol II occupancy and transcription of pre-snRNAs are reduced in *dsp1*, suggesting that DSP1 may also promote snRNA transcription. Supporting this, DSP1 was shown to bind snRNA gene promoters. Furthermore, all available null *dsp1-2, dsp2-1, dsp3-2*, and *cpsf73-1* mutants are embryonically lethal, while *dsp1* and *cpsf73-I* have defective pollen development (Xu et al., 2006; Liu et al., 2016). These observations suggest that the DSP1 complex plays multiple important roles in development.

While DSP4 has sequence similarities with INT9, it does not interact with CPSF73-I, a homolog of INT11 (Liu et al., 2016). Until now, no null *DSP4* mutant allele has been analyzed, and the function of DSP4 in pre-snRNA 3′ maturation and development is still not understood. Here, we show that an amorphic *dsp4-1* mutation impairs growth and male fertility and reduces pre-snRNA transcription and 3′-end processing in Arabidopsis. These phenotypes resembled those of *dsp1*. Interestingly, *dsp1-1 dsp4-1* double mutants are completely male sterile. Pre-snRNA 3′-end processing and transcription is further reduced in *dsp1-1 dsp4-1* relative to *dsp1-1* or *dsp4-1*. Moreover, the cleavage accuracy of the pre-snRNA 3′ end is reduced in *dsp1 dsp4* when compared with the wild type or single mutants. These results, together with the fact that DSP4 interacts with the ARM domain of DSP1 through its β-Casp domain, demonstrate that DSP4 and DSP1 cooperatively promote snRNA transcription and 3′ maturation, and regulate pollen and plant development.

## RESULTS

### The *dsp4-1* Mutation Impairs Development and Male Gametophyte Transmission

**Figure 1.**
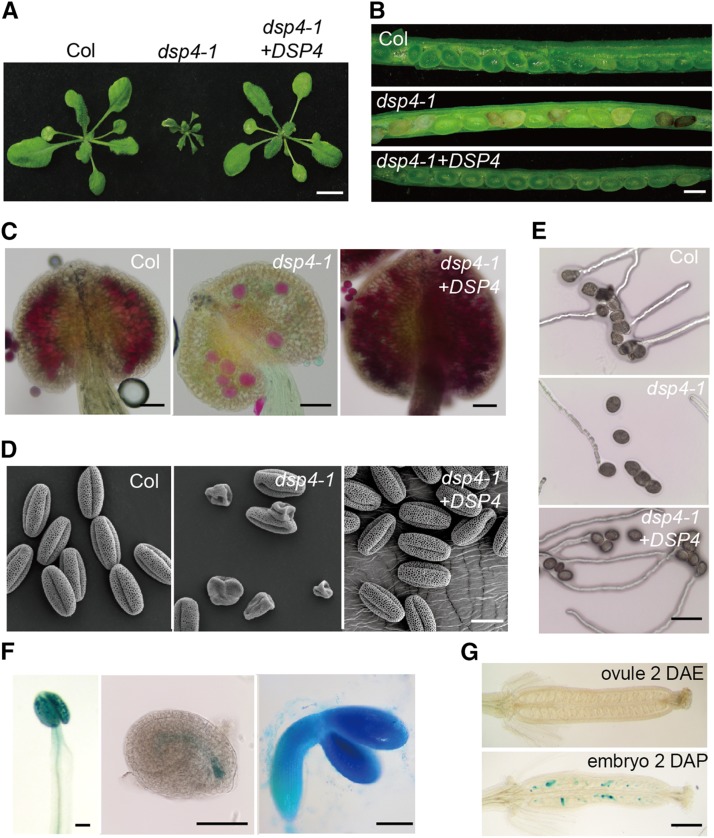
*dsp4-1* causes pleiotropic developmental defects. A, Twenty-five-day-old plants with nine-rosette leaves of Col, *dsp4-1*, and *dsp4-1* harboring the *pDSP4::DSP4-GFP* transgene. B, Developing seeds in siliques of various genotypes. C, Alexander staining of pollen grains in anthers of various genotypes. D, Pollen structures of various genotypes detected by SEM. E, In vitro germination of pollen of various genotypes. Images were obtained at 8 h after incubation in BK medium. F, Histochemical GUS staining of pollen (left), embryo sacs (middle), and embryos (right) of plants containing the *pDSP4::GUS* transgene. G, Histochemical staining of GUS in the siliques of plants containing the *pDSP4::GUS* transgene. DAE, days after emasculation; DAP, days after pollination. Images in E and G are representative of one out of five plants analyzed. Scale bars = 5 mm (A), 1 mm (B), 30 μm (C), 10 μm (D), 20 μm (E), 0.1 mm (F), and 0.5 mm (G).

We previously showed that knockdown of *DSP4* with artificial microRNAs causes developmental defects. To further evaluate the function of *DSP4*, we obtained a *dsp4-1* allele (*SALK_005904*) that contains a transfer DNA insertion in the 10th intron of *DSP4* (Supplemental Fig. S1A). Reverse transcription quantitative PCR (RT-qPCR) analysis using specific primers that span the 10th intron revealed that the *DSP4* transcript levels in *dsp4-1* were greatly reduced relative to that in ecotype Columbia of Arabidopsis (Col; the wild type). Moreover, the size of the *DSP4* transcript was longer in *dsp4-1* than in Col (Supplemental Fig. S1B). Sequencing analysis showed that the increased size of the *DSP1* transcript was caused by retention of the 11th intron that led to a premature stop codon (Supplemental Fig. S1C). Like *DSP4* knockdown lines, *dsp4-1* had delayed growth and fertility; several aborted seeds were detected in *dsp4-1* siliques (Fig. 1, A and B). To demonstrate that *dsp4-1* is responsible for the observed phenotypes, a wild-type copy of the *DSP4* genomic DNA fused with a *GFP* reporter gene driven by its native promoter (*pDSP4::DSP4-GFP*) was transformed into *dsp4-1*. Expression of the wild-type *DSP4* rescued the developmental defects of *dsp4-1*.

The ratio of heterozygous versus wild-type plants in the progeny of *DSP4/dsp4-1* crosses was 1.24:1, which is less than the 2:1 ratio expected by Mendelian inheritance (Supplemental Table S1). This result suggested that, like *DSP1* (Liu et al., 2016), *DSP4* may also reduce gametophyte transmission. To determine whether *dsp4-1* affects female or male gametophyte transmission, we performed reciprocal crosses between *DSP4*/*dsp4-1* and the wild type. When *DSP4/dsp4-1* was used as a pollen donor, the gametophyte penetration of *dsp4-1* was distorted (Supplemental Table S2). In contrast, when *DSP4/dsp4-1* was used as the female parent, *dps4-1* transmitted normally, suggesting that male, but not female, gametophyte transmission was impaired. To verify the influence of *DSP4* on pollen development, we examined pollen viability using Alexander staining (Chen et al., 2016). Only a small number of purple-stained (viable) pollen were found in *dsp4-1* anthers, suggesting that most grains were sterile (Fig. 1C). Furthermore, scanning electron microscopy (SEM) analysis showed that more than half of the pollen grains of *dsp4-1* were shrunken and irregular in shape (Fig. 1D) compared with those of the wild type. Consistently, most *dsp4-1* pollen grains failed to germinate in vitro (Fig. 1E). The *pDSP4::DSP4-GFP* transgene was able to rescue pollen structure, viability, and germination in *dsp4-1* (Fig. 1, C–E), demonstrating that *DSP4* is required for pollen development.

Next, we examined whether the expression pattern of *DSP4* is consistent with its function in pollen development. We generated a transgenic plant expressing a GUS reporter gene under the control of the *DSP4* promoter. Histochemical staining showed that GUS was weakly expressed in leaves and roots, but not in stems, emerging flowers, and unfertilized ovules and eggs (Fig. 1G; Supplemental Fig. S1D). High GUS expression was detected in pollen (Fig. 1F), in agreement with a role for DSP4 in pollen development. GUS was also detected in fertilized eggs and developing embryos (Fig. 1F), suggesting that DSP4 may have an additional role in embryo development. Indeed, we found that aborted *dsp4-1* seeds contained embryos arrested at the globular stage (Supplemental Fig. S1E).

### DSP4 Interacts with the ARM Domain of DSP1 via Its β-Casp Domain

As DSP4 interacts with DSP1, and is required for snRNA 3′-end maturation and development, it is possible that it participates in snRNA processing and development as a component of the DSP1 complex. To evaluate this possibility, we first examined whether DSP4 localized in the nucleus, where snRNA 3′-end processing occurs. We analyzed GFP localization in *dsp4-1* plants harboring the *pDSP4::DSP4-GFP* transgene and found that, indeed, the signals were enriched in the nucleus of pollen and embryo cells (Supplemental Fig. S2, A–C).

**Figure 2.**
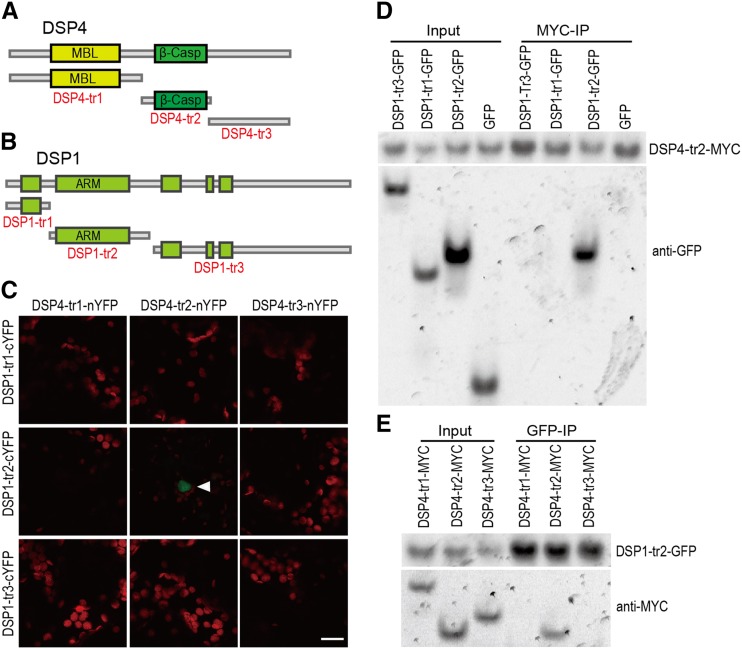
The β-Casp domain of DSP4 and the second ARM cluster of DSP1 mediate the DSP1-DSP4 interaction. A and B, Schemes of full-length and truncated DSP4 (A) and DSP1 (B) used for testing the DSP1-DSP4 interaction. C, The interactions of various forms of DSP4 with truncated DSP1 was detected by BiFC in *N. benthamiana* epidermal cells. Paired DSP1-trs-cYFP and DSP4-trs-nYFP fusion proteins were infiltrated into *N. benthamiana* leaves. The green fluorescence indicates the BiFC signal (originally YFP fluorescence), and the red indicates autofluorescence of chlorophyll. Scale bar = 10 μm. D, Co-IP of DSP4-tr2 with truncated DSP1 proteins. E, Co-IP of DSP1-tr2 with truncated DSP4 truncation proteins. IP was performed using antibodies recognizing MYC (D) or GFP (E). After IP, truncated DSP4-MYC and DSP1-GFP were detected by western blot using antibodies against MYC and GFP, respectively. Input, total protein before IP.

Next, we sought to further confirm and characterize the physical interaction between DSP4 and DSP1 by determining the putative protein domains that mediate the interaction. DSP4 is a homolog of CPSF73, but it is catalytically inactive. It contains a MBL-fold metallo-hydrolase domain (amino acids 101–280), a β-Casp domain (MBL-associated CPSF-73 Artemis SNM1/PSO2; amino acids 374–478), and a C-terminal region. Targeting these three domains, we generated three truncated DSP4 proteins, DSP4-tr1 (amino acids 1–348), DSP4-tr2 (amino acids 333–485), and DSP4-tr3 (amino acids 483–699; Fig. 2A). DSP1 contains three clusters of Armadillo/β-catenin-like repeats (ARM; ∼40 amino acids for each repeat), which provide solvent-accessible surfaces for binding of other substrates. We constructed three truncated versions of DSP1, DSP1-tr1 (amino acids 1–230), DSP1-tr2 (amino acids 224–504), and DSP1-tr3 (amino acids 498–1133), to cover these three ARM clusters (Fig. 2B). We first examined the interaction of truncated DSP1 proteins with truncated DSP4 proteins using bimolecular fluorescence complementation (BiFC). The coexpression of DSP1-tr2-cYFP and DSP4-tr2-nYFP, where no other pairs of truncated proteins were coexpressed (Fig. 2C), resulted in YFP fluorescence signals, suggesting that the second ARM domain of DSP1 and the β-Casp domain of DSP4 are responsible for the DSP1-DSP4 interaction. We next used coimmunoprecipitation (co-IP) to confirm this possibility. We coexpressed, in *Nicotiana benthamiana*, three truncated DSP4 proteins fused with a MYC tag at their C termini, with DSP1-tr2-GFP, or three GFP-fused truncated DSP1 proteins with DSP4-tr2-MYC. After IP, we could detect DSP1-tr2-GFP in DSP4-tr2-MYC and DSP4-tr2-MYC in the DSP1-tr2-GFP precipitates (Fig. 2, D and E), demonstrating that the β-Casp domain of DSP4 and the ARM cluster 2 of DSP1 are essential and sufficient to mediate the DSP1-DSP4 interaction.

### *dsp1-1 dsp4-1* Double Mutants Have Severe Developmental Defects

The interaction of DSP4 with DSP1 raised the possibility that they function as part of a complex in pre-snRNA 3′ maturation and development. To test this, we examined the genetic interaction between *dsp1-1* and *dsp4-1*. We constructed a *dsp1-1 dsp4-1* double mutant by crossing the two single mutants. We were able to identify *dsp1-1 dsp4-1* plants in the F2 population, but with an extremely low ratio of penetration (2:300), which indicates an impaired male and/or female gametophyte transmission. To test this hypothesis, we made reciprocal crosses of *dsp1-1/dsp1-1 DSP4/dsp4-1* or *DSP1/dsp1-1 dsp4-1/dsp4-1* with the wild type. The transmission rate of the double mutations was dramatically reduced relative to that of single mutations when the double mutants were used as pollen donors (Supplemental Table S3). In contrast, this double mutation had a nearly normal penetration rate when the wild type was used as the pollen donor. These results suggest that *dsp1-1 dsp4-1* further reduced male gametophyte transmission rate relative to the single mutations.

**Figure 3.**
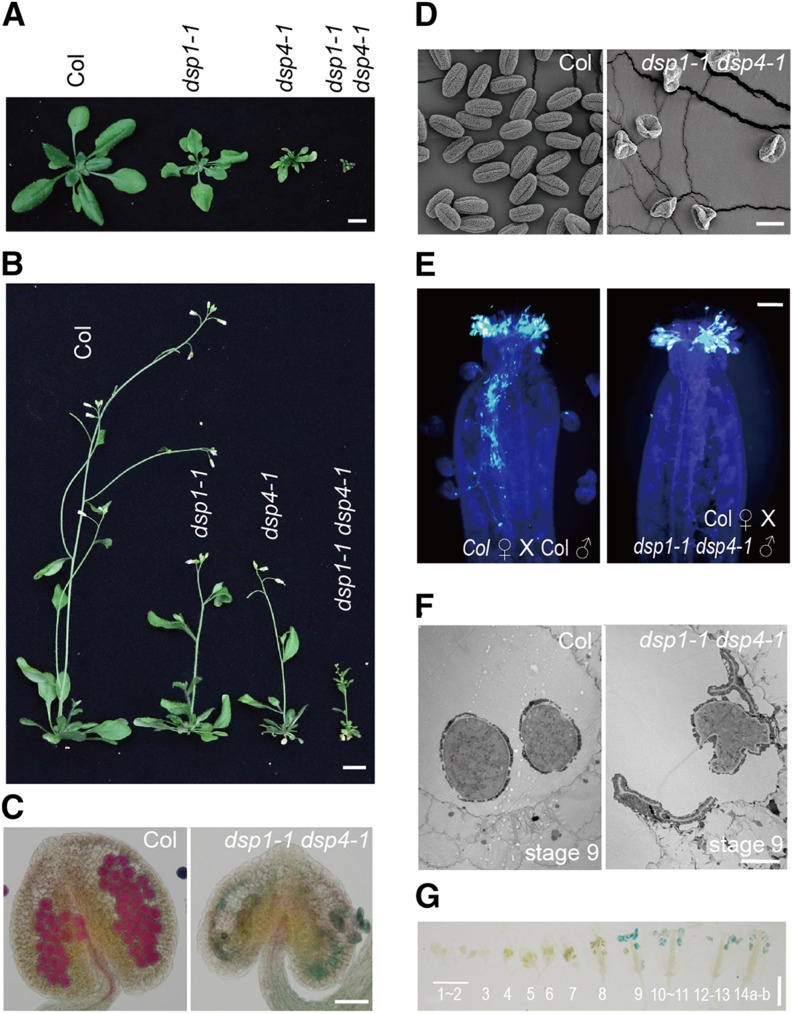
Morphological phenotypes of different genotype plants. A and B, Twenty-eight- (A) and 48-d-old (B) plants of Col, *dsp1-1*, *dsp4-1*, and *dsp1-1 dsp4-1*. C, Alexander staining of pollen grains in anthers of Col and *dsp1-1 dsp4-1*. D, Pollen structures of Col and *dsp1-1 dsp4-1* detected by SEM. E, In vivo pollen germination of Col and *dsp1-1 dsp4-1*. Images were obtained 8 h after fertilization using *dsp1-1 dsp4-1* (right) or Col as a pollen donor (left). F, Transmission electron micrographs showing the microspore structures of Col and *dsp1-1 dsp4-1* at anther stage 9. G, Histochemical GUS staining of inflorescences from *pDSP4::GUS* transgenic plants at different developmental stages. Numbers in the image indicate the developmental stage of flowers. Scale bars = 1.5 cm (A), 3 cm (B), 30 μm (C), 10 μm (D), 0.5 mm (E), 10 μm (F), and 2 mm (G).

Compared with *dsp1-1* or *dsp4-1*, *dsp1-1 dsp4-1* had increased sterility, a strong reduction in size, and an increase in crimped leaves (Fig. 3, A and B). Double mutants also produced almost no viable pollen, as seen by the lack of purple Alexander staining in anthers (Fig. 3C). The grains produced were shrunken, irregular, and adhered together (Fig. 3D; Supplemental Fig. S3, A, E, and F). Moreover, they failed to germinate in vitro (Supplemental Fig. S3, B and F) and in vivo (Fig. 3E; Supplemental Fig. S3G), revealing that *dsp1-1 dsp4-1* failed to produce functional male gametes. These results demonstrate that *DSP1* and *DSP4* act additively in development and pollen growth. To identify the stage of pollen development affected in *dsp1-1 dsp4-1*, we examined pollen morphology in anthers, using transmission electron microscopy. The male gametophytic microspores were divided into 14 developmental stages according to the typical pattern of dicotyledonous plants (Sanders et al., 1999). We collected anthers from wild-type and double-mutant plants at parallel stages, then examined the ultrastructure of male microspores. No significant morphological structure difference was observed before stage 7 between wild-type and *dsp1-1 dsp4-1* anthers (Supplemental Fig. S3C). After stage 8, a large percentage of microspores became vacuolated and then degraded in *dsp1-1 dsp4-1* (Supplemental Fig. S3D). After stage 9, the microspores were completely or partially devoid of cytoplasmic content (Fig. 3F). These results reveal that DSP1 and DSP4 may function after developmental stage 7 of microspores. Consistent with this result, the expression of *DSP4* appeared in the male gametophyte at late stages (stage 9 to the final stage; Fig. 3G), after meiosis, when haploid microspores were generated (Goldberg et al., 1993).

### DSP1 and DSP4 Synergistically Affect the Accumulation of Pre-snRNAs and snRNAs

**Figure 4.**
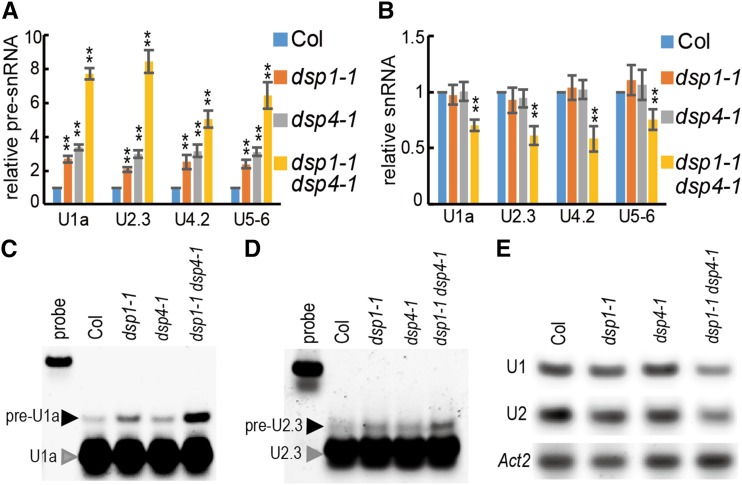
DSP1 and DSP4 synergistically promote snRNA 3′-end maturation. A and B, Accumulation of pre-snRNAs (A) and mature snRNA (B) in various genotypes detected by RT-qPCR. The pre-snRNA or mature snRNA levels were normalized to those of *Actin-2* (*Act2*) and compared with Col (value set at 1). Values are the means of three technical replicates. Bars indicate the sd; *P < 0.05 and **P < 0.01 (Student’s *t* test). Three biological replicates gave similar results. C and D, Accumulation of pre-*U1a* and pre-*U2.3* RNAs in various genotypes detected by the RNase protection assay. Five μg of total RNA were incubated with the U1a or U2.3 RNA probe. Black arrows indicate pre-snRNAs and gray arrows indicate mature snRNAs. E, Abundance of mature *U1* and *U2* snRNAs in various genotypes detected by northern blotting. *Act2* was blotted as the loading control.

Next we examined if DSP1 and DSP4 cooperatively function in snRNA 3′-end maturation. We first tested the accumulation of various pre-snRNAs, including U1a, U2.3, U4.2, and U5-6 in single and double mutants. As expected, the levels of all pre-snRNAs generated from various snRNA loci were uniformly increased in *dsp1-1* and *dsp4-1*, relative to the wild type. Furthermore, all pre-snRNAs were dramatically increased in *dsp1-1 dsp4-1* compared with single mutants (Fig. 4A). A ribonuclease (RNase) protection assay further confirmed this result (Fig. 4, C and D), indicating that DSP1 and DSP4 may synergistically affect pre-snRNA processing. We also examined the effect of DSP1 and DSP4 on the various mature snRNAs. Neither *dsp1-1* nor *dsp4-1* altered the abundance of mature snRNAs (Fig. 4, B and E), consistent with observations in other *dsp* mutants and in *int* metazoan mutants (Tao et al., 2009; Liu et al., 2016). Interestingly, both RT-qPCR and northern-blot analyses showed that the levels of mature snRNAs were reduced in *dsp1-1 dsp4-1* compared with the wild type and single mutants (Fig. 4, B and E). The introduction of DSP4 without the β-Casp domain (*pDSP4::DSP4ΔCasp*) into the *dsp4-1* mutant failed to rescue the phenotypes or pre-snRNA level, thus providing evidence for the importance of the DSP4-DSP1 interaction in snRNA maturation (Supplemental Fig. S4).

### DSP1 and DSP4 Synergistically Act in 3′ End Cleavage of Pre-snRNAs

**Figure 5.**
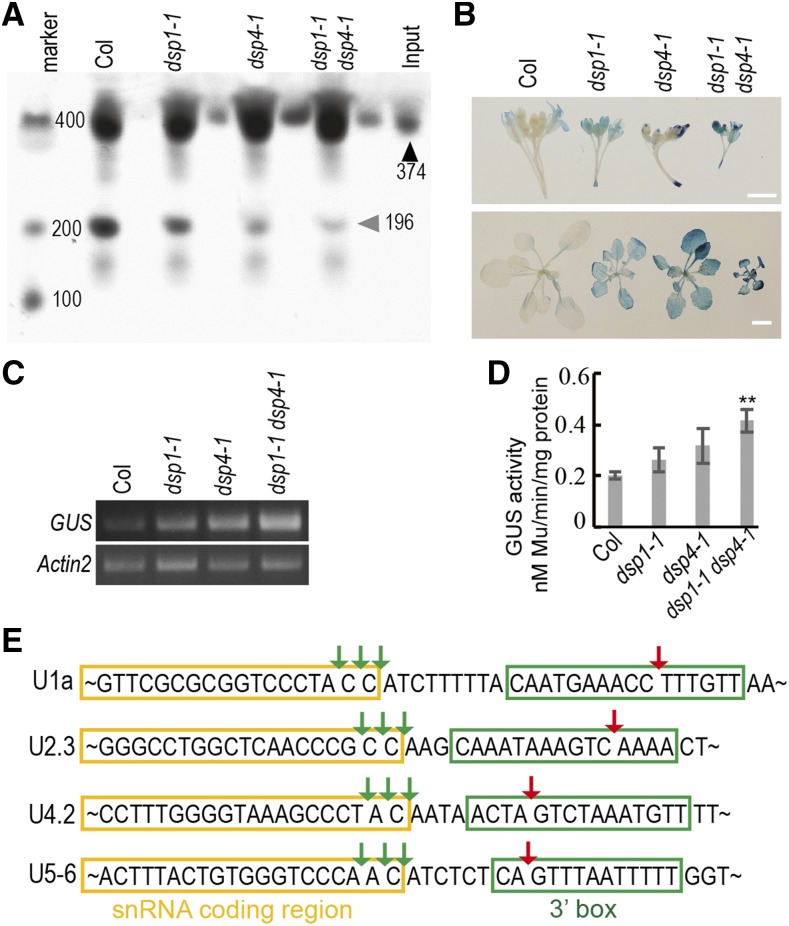
DSP1 and DSP4 cooperatively promote pre-snRNA 3′-end cleavage. A, In vitro 3′-end processing of *pre-U2.3-polyG* in nuclear protein extracts of various genotypes. In vitro transcribed RNAs were labeled at the 5′-end and incubated with the nuclear protein. The black arrow indicates the *pre-U2.3-polyG* input and the gray arrow indicates mature snRNA. B, Histochemical staining of GUS in seedlings (bottom) and inflorescences (top) of Col, *dsp1-1, dsp4-1*, and *dsp1-1 dsp4-1* containing the *pU2::pre-U2-GUS* transgene. Scale bars = 4 mm (top) and 1 cm (bottom). C, *GUS* transcript levels determined by RT-PCR. *Actin2* was amplified as a control. D, In vitro GUS activities of protein inflorescence extracts from various genotypes. Protein extracts were quantified and incubated with 4-methylumbelliferyl β-d-glucuronide and the reaction was stopped by adding Na_2_CO_3_ after 10 min. The 4-methylumbelliferone (Mu) products were measured with a spectrophotometer at 595 nm. Values are the means of three technical replicates. Bars = sd, **P < 0.01 (Student’s *t* test). E, The 3′-end cleavage site of pre-snRNAs in *dsp1-1 dsp4-1*. Green arrows indicate correct cleavage sites. Red arrows indicate improper cleavage sites.

The increased accumulation of pre-snRNAs and the reduced abundance of snRNAs in *dsp1-1 dsp4-1* relative to single mutants suggest that DSP1 and DSP4 additively affect 3′-end cleavage of pre-snRNAs. To test this possibility, we examined the effect of *dsp1-1 dsp4-1* on in vitro 3′-end cleavage of *pre-U2.3* snRNA. The [P^32^]-labeled *pre-U2.3* snRNA, with a *poly-G* tail that prevents 3′-end trimming, was incubated with nuclear protein extracts from inflorescences of Col, *dsp1-1*, *dsp4-1*, and *dsp1-1 dsp4-1* plants. After a 60-min reaction, RNAs were purified and resolved on a PAGE gel to examine the production of mature snRNAs. As previously reported, the amount of U2 RNAs was reduced in *dsp1-1* and *dsp4-1* compared with the wild type. Relative to *dsp1-1* or *dsp4-1,* the level of U2 was further reduced in *dsp1-1 dsp4-1* (Fig. 5A), suggesting a cooperative effect of DSP1 and DSP4 on pre-snRNA 3′-end processing.

Next, we used an in vivo GUS reporter system to validate the synergistic effect of DSP4 and DSP1 on pre-snRNA processing. In this system, a GUS reporter gene was inserted downstream of the 3′ box within the pre-U2 gene containing the promoter, the coding region, and the 3′ box (*pU2::pre-U2-GUS*). This system has been used to monitor the effect of *dsp1-1* on pre-snRNA 3′ cleavage efficiency, because GUS protein levels are inversely proportional to the cleavage upstream of the 3′ box within pre-U2-GUS (Liu et al., 2016). We crossed three independent stable transgenic wild-type lines harboring *pU2::pre-U2-GUS* with *dsp1-1*, *dsp4-1*, and *dsp1-1 dsp4-1*, in parallel, and monitored GUS expression in these four genotypes. As expected, GUS levels were increased in *dsp1-1* and *dsp4-1* relative to the wild type due to impaired 3′-end maturation of *pre-snRNA*s (Fig. 5B), with three biological replicates giving similar results. We also observed that GUS expression levels and GUS activity were further increased in *dsp1-1 dsp4-*1 compared with single mutants (Fig. 5, C and D). These results demonstrate that DSP4 is essential, and acts cooperatively with DSP1, in pre-snRNA 3′-end cleavage.

We further examined the cleavage sites of mature snRNAs. Total RNA was attached with RNA adaptors, then reverse transcribed to single DNA strands. The randomly selected snRNAs from the U1, U2, U4, and U5 gene families were cloned using nested primers, then sequenced to examine the cleavage sites. In the wild type, *dsp1-1*, and *dsp4-1*, cleavage occurred upstream of the 3′ box (Table 1). However, in *dsp1-1 dsp4-1*, a portion of snRNAs (6%–10%) was miscleaved at the 3′ box (Fig. 5E; Table 1). This result reveals that the accurate 3′ cleavage of pre-snRNA requires the cooperative action of DSP1 and DSP4.

**Table 1.** Ratio of miscleaved snRNAs in single and double mutants The RT-qPCR products of different snRNAs from *dsp1-1 dsp4-1* were cloned into the p*MD-18T* vector, and 192 randomly selected clones for each snRNA were sequenced. The miscleaved ratio (MR) was calculated as the number of each cleaved type of snRNA clone divided by the total analysis number (192). The normal cleaved ratio of snRNAs (NR) includes three types of cleavage site (the −1 site, the middle site, and the +1 site).

snRNA	Col		*dsp1-1*		*dsp4-1*		*dsp1-1 dsp1-1*	
	NR	MR	NR	MR	NR	MR	NR	MR
*U1a*	28.5:39.6:31.9	0	26.7:40.3:33.0	0	27.3:40.3:32.4	0	21.7:33.4:38.6	6.3
*U2.3*	25.2:40.5:34.3	0	26.7:41.7:31.6	0	27.0:42.3:30.7	0	19.6:35.2:38.2	7.0
*U4.2*	30.2:41.2:28.7	0	29.3:39.4:31.3	0	31.6:38.0:30.4	0	25.2:34.8:33.2	6.8
*U5-6*	20.4:46.8:32.8	0	24.3:47.6:28.1	0	22.9:48.4:28.7	0	19.2:32.5:38.8	9.5

### DSP4 Impairs the Occupancy of Pol II and DSP1 on snRNA Loci

**Figure 6.**
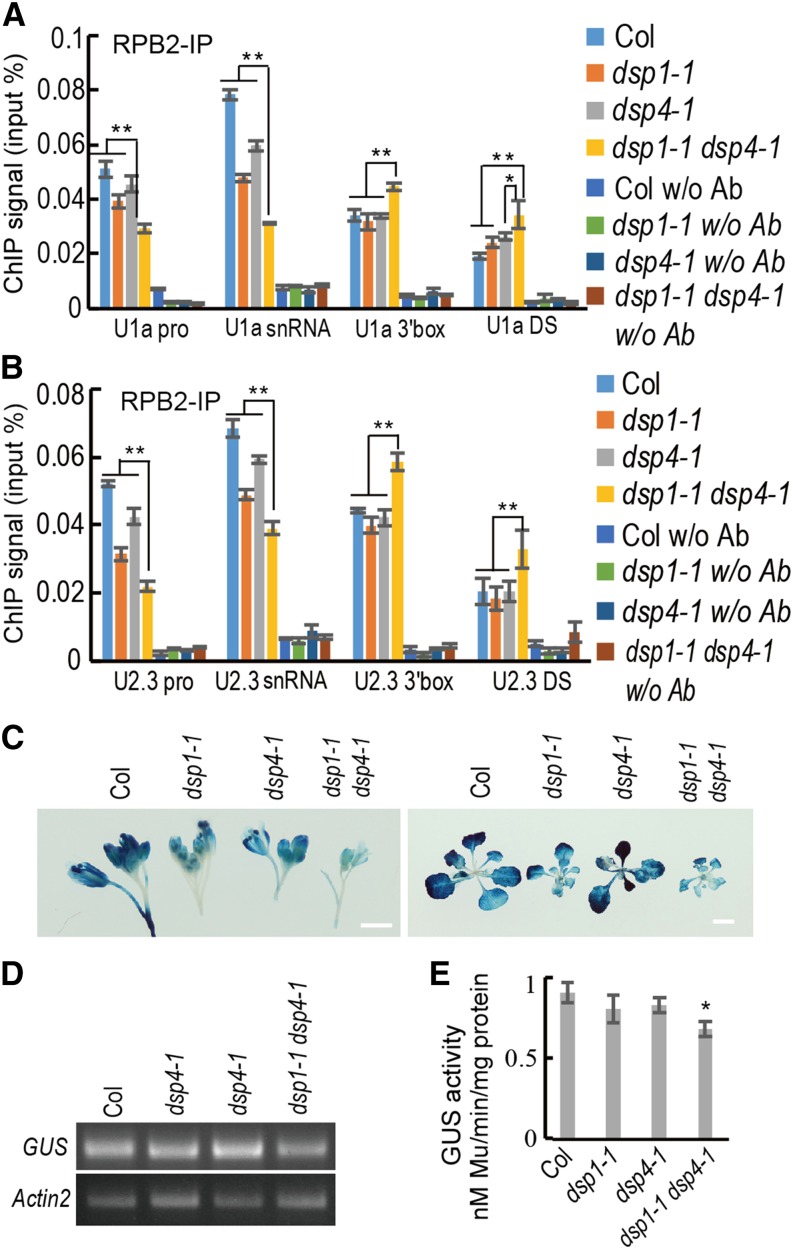
DSP1 and DSP4 synergistically promote pre-snRNA transcription. A and B, Occupancy of Pol II at various sites of the *U1a* (A) and *U2.3* (B) snRNA loci in Col, *dsp1-1*, *dsp4-1*, and *dsp1-1 dsp4-1* detected by ChIP. pro, promoter; DS, downstream region of snRNAs. Values are means of three technical replicates. Bars indicate the sd; *P < 0.05, **P < 0.01 (Student’s *t* test). C, GUS histochemical staining of 28-d-old seedlings (left) and 48-d-old inflorescences (right) in Col, *dsp1-1*, *dsp4-1*, and *dsp1-1 dsp4-1*, containing the *pU2::pre-U2m-GUS* transgene. *m*, mutated 3′ box. Scale bars = 4 mm (left) and 1 cm (right). D, *GUS* transcript levels determined by RT-PCR. *Actin2* was amplified as a control. E, In vitro GUS activity in inflorescence protein extracts from various genotypes. The quantified total protein extracts were incubated with 4-methylumbelliferyl β-d-glucuronide, the reaction was stopped at 10 min, and 4-methylumbelliferone was measured using a spectrophotometer. Values are means of three technical replicates. Bars = sd, *P < 0.05 (Student’s *t* test).

We have shown that *dsp1-1* affects the occupancy of Pol II in the promoter and coding regions of snRNAs but not in the 3′ box. Since DSP4 acts synergistically with DSP1, we investigated whether DSP4 and DSP1 also cooperatively influence Pol II occupancy at various regions of pre-snRNAs, using the chromatin immunoprecipitation (ChIP) assay with antibodies recognizing RPB2, the second largest subunit of Pol II. We also included the downstream region of the 3′ box in the experiment because it has been shown that impaired snRNA 3′ maturation can lead to pre-snRNA 3′-end extension (Fukudome et al., 2017). Like *dsp1-1*, *dsp4-1* also reduced Pol II occupancy at the promoters and coding regions of *U1a* and *U2.3*, but not at the 3′ box and downstream regions (Fig. 6A). In contrast, Pol II had comparable occupancy levels at the *Actin2* loci in plants of all genotypes, or was not associated with the *Pol II C1* loci, which is an intergenic DNA region (Supplemental Fig. S5). These results suggest that, like DSP1, DSP4 is required for proper occupancy of Pol II at snRNA loci. Compared with single mutants, *dsp1-1 dsp4-1* further reduced the association of Pol II with the promoters and coding regions of U1a and U2.3. A higher occupancy of Pol II at the 3′ box and the downstream region was also observed in *dsp1-1 dsp4-1* relative to other genotypes. These data suggest that DSP1 and DSP4 may synergistically impact Pol II accumulation at snRNA genes.

The reduced Pol II occupancy at the promoter and coding regions of snRNAs in single or double mutants indicates that DSP1 and DSP4 may also positively regulate snRNA transcription. To test this possibility, we monitored pre-U2 transcription using a *pU2::pre-U2m-GUS* transgene. Since the *pre-U2m-GUS* RNA contains a mutated 3′ box and cannot be cleaved there, its transcript levels or GUS levels in transgenic plants will not be affected by 3′ cleavage, and thus, it can be used to monitor pre-snRNA expression. We transformed this transgene into the wild type, crossed three independent stable transgenic lines into *dsp1-1*, *dsp4-1*, and *dsp1-1 dsp4-1*, in parallel, and examined the expression levels of GUS and *pre-U2m-GUS* in lines with the *pre-U2m-GUS* transgene, in all genotypes. GUS staining, RT-qPCR, and GUS activity analyses revealed elevated levels of *pre-U2m-GUS* in *dsp1-1, dsp4-1*, and *dsp1-1 dsp4-1* compared with the wild type (Fig. 6, C–E), with three biological replicates giving similar results. In addition, the levels of pre-U2m-GUS were lower in *dsp1-1 dsp4-1* than in *dsp1-1* or *dsp4-1*. These data are consistent with the ChIP results and show that DSP1 and DSP4 additively promote snRNA transcription.

## DISCUSSION

We have previously shown that the DSP1 complex is responsible for snRNA 3′ maturation (Liu et al., 2016). However, the precise roles of DSP components in this process, and how these proteins coordinately catalyze snRNA 3′ maturation, remained unknown. In this study, we showed that DSP4 is an essential component of the DSP complex, evidenced by the reduced pre-snRNA processing efficiency in *dsp4-1*. Moreover, DSP4 promotes snRNA transcription, given its positive impact on Pol II occupancy at the promoters of snRNAs and accumulation of pre-snRNAs. Our results also demonstrated that DSP1 and DSP4 synergistically influence CPSF73-I activity in snRNA 3′-end cleavage, given the reduced pre-snRNA cleavage efficiency and accuracy in *dsp1 dsp4* double mutants relative to *dsp1* or *dsp4*.

What is the function of DSP4 in pre-snRNA 3′-end cleavage? It has been known that CPSF73 and its homolog CPSF100 form a heterodimer, which is required for the endonuclease activity that cleaves pre-mRNA during the polyadenylation process (Kolev et al., 2008; Sullivan et al., 2009). Like CPSF100, DSP4 is a catalytically inactive endonuclease of the MBL/β-CASP family. By analogy, DSP4 could act with CPSF73-I to form a functional endonuclease for pre-snRNA 3′-end cleavage. However, DSP4 does not interact with CPSF73-I. Instead, DSP4 interacts with DSP1, which also interacts with CPSF73-I. Moreover, these three proteins coexist in a complex (Liu et al., 2016). These results raise the possibility that DSP1 mediates the association between DSP4 and CSPF73-I, which in turn facilitates CPSF73-I activity. This model predicts that DSP1 acts as a scaffold for the assembly of an active endonuclease for pre-snRNA cleavage. Supporting this model, the DSP1 homolog in metazoans, INT4, interacts with and stabilizes the INT9-INT11 heterodimer, which is required for efficient pre-snRNA cleavage (Albrecht et al., 2018). Based on this model, we expected that *dsp1 dsp4* would have an impact on snRNA processing similar to that observed for *dsp1* or *dsp4*. However, we observed a synergistic effect of DSP1 and DSP4 on pre-snRNA processing activity. In addition, we found that the cleavage accuracy of pre-snRNAs by CPSF73-I is impaired in *dsp1 dsp4*. These results suggest that DSP1 and/or DSP4 have additional roles in regulating CPSF73-I cleavage efficiency and in defining cleavage sites for CPSF73-I. In metazoans, INT does not promote snRNA transcription, but in plants, DSP1 and DSP4 appear to play cooperative roles in recruiting Pol II to the promoters of snRNA genes. These results suggest that the mechanisms promoting snRNA transcription differ between plants and metazoans, despite the common need for the snRNA activating protein complex (Guiro and Murphy, 2017).

DSP4 also plays important roles in development. Interestingly, we find that DSP4 is highly expressed in pollen but not in ovules. In agreement, DSP4 is required for male germline development. However, the levels of mature snRNAs in *dsp4* are comparable with those in the wild type, suggesting that DSP4 has functions other than in snRNA biogenesis. DSP1 and DSP4 appear to have a synergistic effect on general and male germline development, given the increased severity of phenotypes in *dsp1 dsp4* relative to single mutants. Although it is possible that the enhanced developmental defects of *dsp1 dsp4* are due to reduced snRNA levels, other possibilities exist. Both *DSP1* and *DSP4* mutations affect male gametophyte development, while *srd2*, a mutant with defective snRNA transcription, was affected exclusively in the female gametophyte (Ohtani et al., 2008). This opposite phenotype also supports the position that DSP1 and DSP4 act on snRNA maturation and germline development through parallel pathways. It is possible that DSP1 and DSP4 have cooperative effects on the metabolism of RNAs other than snRNAs. Indeed, INT could affect transcription termination of some mRNAs and the biogenesis of enhancer RNAs in metazoans (Gardini et al., 2014; Stadelmayer et al., 2014; Lai et al., 2015; Skaar et al., 2015). Moreover, 3′ extended transcription of snRNAs can produce protein-coding transcripts from downstream snRNA loci (Fukudome et al., 2017). It is tempting to speculate that impaired 3′-end cleavage in *dsp1 dps4* results in extended transcription of snRNAs and biogenesis of protein-coding transcripts, which may disrupt normal development.

## MATERIALS AND METHODS

### Plant Materials

All transfer DNA insertion mutants (*dsp4-1* SALK_005904 and *dsp1-1* SALK_036641) were obtained from the Arabidopsis Biological Resources Center (https://abrc.osu.edu). All mutants are in the Columbia (Col) genetic background.

### Plasmid Construction

The 1.97 kb promoter region of *DSP4* was cloned into *pENTR/SD/D-TOPO* and subsequently cloned into *pGWB433* (Nakagawa et al., 2007) to generate the *pDSP4::GUS* vector. The genomic fragments of *DSP4* containing the promoter and coding regions were PCR amplified and cloned into *pGWB4* to generate the *pDSP4::DSP4-GFP* vector. The truncated gene sequences of *DSP1* and *DSP4* were cloned into *pGWB4* and *pGWB17* to generate *DSP1-trs-GFP* and *DSP4-trs-MYC* vectors, respectively. The primers used for plasmid construction are listed in Supplemental Table S4.

### Histochemical GUS Staining

For GUS staining, seedlings, inflorescence, or embryos dissected from immature seeds were directly incubated overnight in the GUS staining buffer, in the dark and at 37°C. After removing the chlorophyll in 70% (v/v) ethanol, GUS staining was observed under an Olympus light microscope.

### Pollen Viability and Pollen Growth Assays

Alexander staining was used to examine pollen viability as previously described by Xu et al. (2015). In vitro pollen growth and in vivo pollen germination assays were performed as described before in Chen et al. (2016). Briefly, for the in vitro pollen growth assay, mature anthers were collected and vortexed in Brewbaker and Kwack (BK) lipid medium. Then, the deposited pollen was spread on BK medium for 8 h, and observed by microscopy.

### Observation of Pollen Grain Structures

Ultrastructures of pollen grain were examined according to Wang et al. (2017). Briefly, anthers at various developmental stages were fixed, dehydrated, embedded in resin (Epon812), and cut to semifine sections (0.6-0.8 μm) with a Leica microtome for optical analyses. Serial sections were stained with toluidine blue. The slides were observed on an Olympus BX51 microscope. To observe the ultrastructure, the same blocks used for the optical microscope observations were cut to ∼80-nm sections with a diamond knife. The sections were collected on grids and sequentially stained with uranyl acetate and lead citrate. Following contrast and washing, the superthin sections were observed by transmission electronic microscopy (JEOL).

### U2.3 Pre-snRNA In Vitro Processing Assay

In vitro processing assays of *pre-U2.3-polyG* were performed as described before in Uguen and Murphy (2004) and Liu et al. (2016). Briefly, *pre-snRNA-polyG* was generated by in vitro transcription using the T7 RNA polymerase. RNA substrates were purified using an 8% (w/v) polyacrylamide gel with 8 m urea, then 5′ labeled with [P^32^] using T4 Polynucleotide Kinase (T4 PNK). Then, the *pre-snRNA-polyG* RNA was incubated for 60 min with 2 μg nuclear proteins extracted from various plants, in a reaction buffer containing 10 mm HEPES, pH 7.9, 50 mm KCl, 10% (v/v) glycerol, 20 mm creatine phosphate, 3 mm MnCl_2_, 3% (w/v) polyethylene glycol, and 1 mm dithiothreitol. Following the reaction, RNAs were extracted, purified, and resolved on 5% (w/v) PAGE gels with 8 m urea. Radioactive signals were detected with a PhosphorImager (GE Typhoon).

### RNase Protection Assay

Synthesized antisense RNA was labeled with [P^32^] using T4 PNK. Five micrograms of total RNA extracted from inflorescences using the Trizol Reagent were incubated with radiolabeled RNA probes. RNase protection assays were performed using RNase T1 and RNase A as previously described by Carey et al. (2013). After the reactions, the final protected RNAs were separated in a 6% PAGE gel containing 8 m urea. Radioactive signals were detected with a PhosphorImager.

### Pre-snRNA Cleavage Site Analysis

The 3′-end cleavage sites of pre-snRNAs were analyzed according to Liu et al. (2016). Briefly, RNA samples were dephosphorylated, and then ligated to a 3′ RNA adaptor. The ligation products were further purified with phenol/chloroform, ethanol precipitated, and then used as templates for RT (primer sequences are in Supplemental Table S4). The RT products were used as templates for nest-PCR amplification. The resulting PCR products were cloned into *pGEM-T Easy* Vector for sequencing.

### ChIP Assay

ChIP assays with anti-Pol II were performed as described before by Liu et al. (2016). Anti-RPB2 (Abcam) antibodies were used for IP. Enrichment of the target DNA loci relative to input were measured by qPCR with three biological replicates. The primers used in ChIP-PCR are listed in Supplemental Table S4.

### BiFC and the Co-IP assay

*DSP4* and *DSP1* fragments were fused at their N termini with nYFP (*pEarleyGate201-YN*) and cYFP (*pEarleyGate202-YC*), respectively. GV3101 *Agrobacterium* cells transformed with different combinations of *DSP1-trs a*nd *DSP4-trs* were infiltrated into the leaves of *Nicotiana benthamiana*. Epidermal cells were examined with confocal microscopy (Leica TCS SP5). For the co-IP assay, a mixture of *Agrobacterium* containing different combinations of DSP1-trs-GFP and DSP4-trs-MYC were expressed in N. *benthamiana* leaves and then used for IP assays as described before by Zhang et al. (2013). The IP proteins were analyzed by western blot.

### Statistical Analyses

The statistical analyses, including Student’s *t* test, were performed by Excel 2010 software. The qPCR for each sample was replicated three times unless noted otherwise, the average values of 2-ΔCT were used to determine the differences, and the data were expressed as the mean ± sd. A significant difference was considered at *P* < 0.05 and extremely significant at *P* < 0.01.

### Accession Numbers

Sequence data from this article can be found in the Arabidopsis Genome Initiative or GenBank/EMBL data libraries under the following accession numbers: AT4G20060 (DSP1), AT3G07530 (DSP4), AT1G61010 (CPSF73-I).

### Supplemental Data

The following supplemental materials are available.

**Supplemental Figure S1.** Analyses of *dsp4-1* and the expression pattern of *DSP4*.
**Supplemental Figure S2.** Subcellular localization of DSP4.
**Supplemental Figure S3.** *dsp1-1 dsp4-1* double mutants have complete male sterility.
**Supplemental Figure S4.** The importance of the DSP4-DSP1 interaction.
**Supplemental Figure S5.** Pol II occupancy at *Act2* and *Pol II C1* loci.
**Supplemental Table S1.** Segregation ratio in the offspring of *DSP4/dsp4-1* plants.
**Supplemental Table S2.** Analysis of gametophyte transmission in heterozygous plants by reciprocal crosses.
**Supplemental Table S3.** Analysis of gametophyte transmission in double mutants by reciprocal crosses.
**Supplemental Table S4.** List of primers used in this study.
